# Biased Brownian Motion of KIF1A and the Role of Tubulin’s C-Terminal Tail Studied by Molecular Dynamics Simulation

**DOI:** 10.3390/ijms22041547

**Published:** 2021-02-04

**Authors:** Yukinobu Mizuhara, Mitsunori Takano

**Affiliations:** Department of Pure and Applied Physics, Waseda University, Okubo 3-4-1, Sinjuku-Ku, Tokyo 169-8555, Japan; yknb-bnjv10.2@ruri.waseda.jp

**Keywords:** Brownian ratchet, kinesin, microtubule, electrostatic interaction, axonal transport

## Abstract

KIF1A is a kinesin family protein that moves over a long distance along the microtubule (MT) to transport synaptic vesicle precursors in neurons. A single KIF1A molecule can move toward the plus-end of MT in the monomeric form, exhibiting the characteristics of biased Brownian motion. However, how the bias is generated in the Brownian motion of KIF1A has not yet been firmly established. To elucidate this, we conducted a set of molecular dynamics simulations and observed the binding of KIF1A to MT. We found that KIF1A exhibits biased Brownian motion along MT as it binds to MT. Furthermore, we show that the bias toward the plus-end is generated by the ratchet-like energy landscape for the KIF1A-MT interaction, in which the electrostatic interaction and the negatively-charged C-terminal tail (CTT) of tubulin play an essential role. The relevance to the post-translational modifications of CTT is also discussed.

## 1. Introduction

KIF1A, a kinesin-3 family protein, is a molecular motor that moves on the microtubule (MT) over a long distance toward the plus-end of MT. Along the axonal MT in neurons, KIF1A transports synaptic vesicle precursors, which contain synaptic vesicle proteins, indispensable for synaptogenesis [1,2], while KIF1A mutants lead to severe neuronal degeneration, synaptic dysfunction, and neuropathies [2,3,4]. In hippocampal neurons, KIF1A transports dense-core vesicles that contain neurotrophins [5], which are involved in the hippocampal synaptogenesis and learning enhancement in an enriched environment [6]. Interestingly, KIF1A is able to move along the MT unidirectionally in the monomeric form [1], which is in contrast to the conventional kinesin (kinesin-1) that moves along the MT in the dimeric form. While the way a dimeric kinesin moves along the MT can be clearly visualized by the hand-over-hand mechanism [7], the way a monomeric KIF1A moves along the MT is elusive. Notably, single molecule experiments demonstrated that the movement of a monomeric KIF1A along the MT is well characterized by biased Brownian motion [1,8,9], and the bias toward the plus-end of the MT is generated when KIF1A binds to the MT [9]. It was also shown that the electrostatic interaction between the positively-charged loop (K-loop) of KIF1A and the negatively-charged C-terminal tail (CTT) of tubulin, called the E-hook, plays an essential role in one-dimensional Brownian motion along the MT [8]. The importance of one-dimensional Brownian motion and the electrostatic interaction between the K-loop and the CTT has been indicated in the long-distance movement of the dimeric form of KIF1A as well [10,11].

Seeking a physical explanation for biased Brownian motion, computational studies to simulate the binding of KIF1A to the MT have been done based on the three-dimensional structures [12,13], but the physical basis for the biased Brownian motion of KIF1A has not yet been firmly established. In particular, the role of the CTT in biased Brownian motion has not been fully explored. Recently, the role of the CTT has attracted much attention from the viewpoint of the “tubulin code” as well [14,15]; the interaction of the CTT with MT-binding proteins, including KIF1A, is regulated by the post-translational modifications of the CTT, in particular by polyglutamylation, which strengthens the net negative charge of the CTT [16,17].

In this study, we conducted *in silico* single molecule experiments, a set of molecular dynamics simulations, to study the binding of KIF1A to the MT, as was done previously for the actomyosin molecular motor where a single myosin molecule exhibits unidirectional Brownian motion along an actin filament during the weak-to-strong binding transition to actin [18]. By conducting a number of binding simulations (2400 runs), we observed statistically significant unidirectional movement of KIF1A along the MT in the course of binding to MT. By analyzing the KIF1-MT interaction, we found that the energy landscape has a ratchet-like profile along the MT, where the negative charge of the CTT plays a key role.

## 2. Results

### 2.1. Biased Brownian Motion of KIF1A along the MT

**Figure 1:**
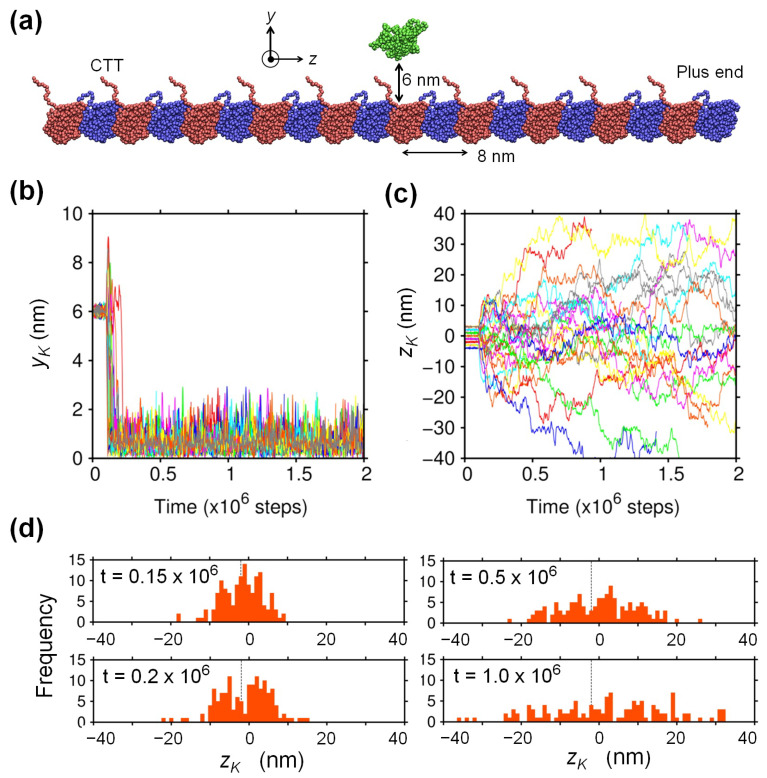
Binding of KIF1A to the microtubule (MT). (**a**) The system we employed in this study is composed of a monomeric KIF1A (green) and a single protofilament of the MT with 20 tubulins (blue: α tubulin; red: β tubulin). KIF1A was initially moved from the original bound position in the EM structure [20] by +6 nm along the *y*-axis (the mass center of the bound position of KIF1A is used as the coordinate origin). (**b**,**c**) Time courses for *y*- and *z*-positions of the mass center of KIF1A, denoted by $y_{K}$ and $z_{K}$, respectively, are shown. Sample trajectories (randomly selected 24 runs) where binding of KIF1A to the MT was observed are displayed. The time course data are shown until KIF1A dissociated from the MT. Note that the unit time step in this study corresponds to 3.3 × 10${}^{-5}$ ns and that the translational motion of KIF1A was enhanced by ∼100-fold because we employed a low viscosity (see the Methods). For equilibration, KIF1A was restrained at each initial position for the first $0.1\times 10^{6}$ steps before starting the observation of the binding. (**d**) Time evolution of $z_{K}$ for the ensemble of KIF1A that started from $z_{K}=-2$ nm (142 runs); the distributions at t= 0.15, 0.2, 0.5, and 1.0 × 10${}^{6}$ steps are shown. CTT, C-terminal tail.

To study the binding of monomeric KIF1A to the MT by molecular dynamics (MD) simulation, we employed a minimal system constituted by a single monomeric KIF1A and a single MT protofilament containing 20 tubulins (Figure 1a). It is known that a single MT protofilament provides the minimal track for kinesin [19]. We used the KIF1A-MT complex structure solved by cryo-electron microscopy (EM) [20] as the base structure of this study. In this study, the protofilament was set in parallel to the *z*-axis and was fixed in space. The positive direction of *z* corresponds to the plus-end direction of the MT, and the origin of the coordinate system was placed at the mass center of KIF1A in the MT-bound position in the EM structure. The *y*-axis was set perpendicular to the surface of the MT, and we initially moved KIF1A away from the MT by +6 nm along the *y*-axis. We prepared eight initial *z*-positions of KIF1A with equal spacing of 1 nm so that they cover the entire 8 nm period of the protofilament. For each of the eight initial positions, we conducted 300 binding simulations and observed the *y*- and *z*-positions of the mass center of KIF1A, denoted by $y_{K}$ and $z_{K}$, respectively.

In Figure 1b,c, the time courses of $y_{K}$ and those of $z_{K}$ are shown, respectively, for the MD trajectories where the binding of KIF1A to MT was observed. We judged that binding of KIF1A to MT occurred when $z_{K}$ became less than 1 nm and excluded the trajectories where KIF1A moved far from the MT ($z_{K}>10$ nm). Accordingly, we observed the binding of KIF1A to the MT in 1218 runs (out of a total of 2400 runs). Looking first at the time courses of $y_{K}$ (Figure 1b), we can see that KIF1A directly approaches the MT in most cases, which indicates that KIF1A experiences sufficiently strong and long-range attraction to the MT. From the time courses of $z_{K}$ (Figure 1c), we can recognize that KIF1A moves along the MT back and forth in a stochastic manner, like one-dimensional Brownian motion, with some spatially-trapped regions. See also the distribution of $z_{K}$ and its time evolution (Figure 1d).

**Figure 2:**
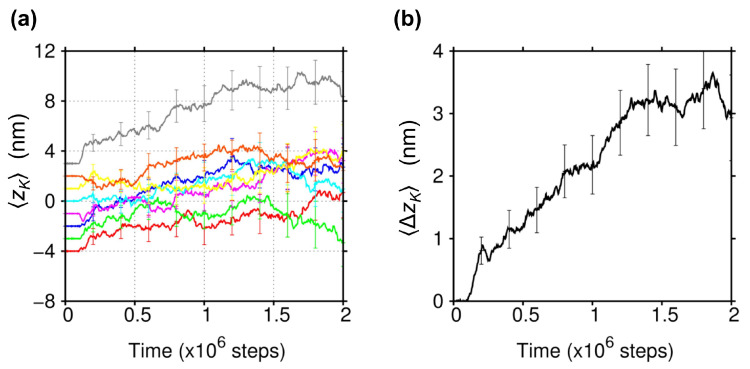
Ensemble behavior of KIF1A movement along the MT. (**a**) Average time-course of $\langle z_{K}\rangle$ started from each of the eight initial positions ($z_{K}\left(0\right)=-4,-3,-2,-1,0,+1,+2,+3$ nm, colored in red, green, blue, magenta, cyan, yellow, orange, and gray, respectively). For averaging, we excluded the trajectories where KIF1A moved far from the MT ($z_{K}>10$ nm). Binding was judged to occur when $z_{K}<1$ nm. The time course data before unbinding were used to calculate the average. (**b**) Ensemble average of the *z*-displacement of KIF1A as a function of time, $\langle \Delta z_{K}\left(t\right)\rangle \equiv \langle z_{K}\left(t\right)-z_{K}\left(0\right)\rangle$, obtained from the trajectories where KIF1A binding was observed (total 1218 runs). Error bars indicate the statistical uncertainty (the standard error of the mean) at the 68% confidence interval.

Furthermore, by taking the ensemble average of all the binding trajectories that started from each of the eight initial *z*-positions, it was found that Brownian motion along the MT is overall biased toward the plus-end (Figure 2a). The bias of Brownian motion toward the plus-end is more clearly seen by averaging out the dependence of the initial *z*-positions. In Figure 2b, we show the average displacement of KIF1A along the MT, $\langle \Delta z_{K}\left(t\right)\rangle \equiv \langle z_{K}\left(t\right)-z_{K}\left(0\right)\rangle$, where the average was taken over the eight initial *z*-positions using all 1218 binding trajectories. Then, statistically significant displacement of $\langle \Delta z_{K}\rangle ∼3$ nm can be seen, which is in coincident with the bias of ∼3 nm observed in the single molecule experiment by Okada et al. [9]. Note that the bias toward the plus-end was observed as well when KIF1A was allowed to rotate freely about its central *y*-axis even though the distribution of $z_{K}$ became larger due to the enhanced orientational motion of KIF1A (see Supplementary Figure S1). Note also that the MT protofilament employed in our study is so long that the ends of the protofilament cannot affect the behavior of KIF1A.

### 2.2. Ratchet-Like Energy Landscape for KIF1A-MT Interaction

**Figure 3:**
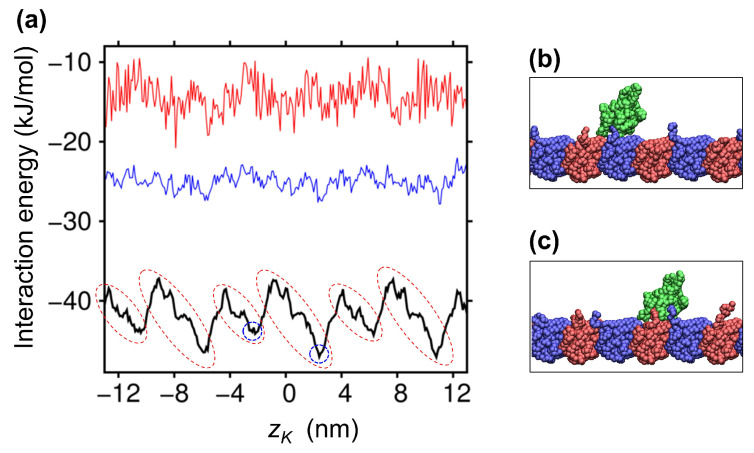
(**a**) The average interaction energy between KIF1A and the MT is shown as a function of $z_{K}$ (black). Broken red circles indicates the slopes toward the plus-end of the MT. Average interaction energies at greater salt strengths are also displayed (blue: 150 mM (λ=0.78); red: 200 mM (λ=0.67)). (**b**,**c**) Snapshot structures at the energy minima, located at $z_{K}∼-2$ nm (**b**), and at $z_{K}∼2$ nm (**c**), which are designated by broken blue circles in (**a**).

To elucidate where the bias of Brownian motion originates, we analyzed the interaction energy between KIF1A and the MT. In Figure 3a, we show the energy landscape for the KIF1A-MT interaction as a function of $z_{K}$, which was obtained by averaging the potential energies for the KIF1A-MT interaction at a given $z_{K}$. The energy landscape, depicted by the black line in Figure 3a, exhibits an 8-nm periodicity, reflecting the periodicity of the MT protofilament. Furthermore, we can recognize that the energy landscape presents a ratchet-like profile, with the gentler slope toward the plus-end (see the regions indicated by broken red circles). The gentler and hence wider slope toward the plus-end means that when KIF1A binds to the MT, it has a greater probability to fall within this wider slope region than in the narrower slope toward the minus-end. Therefore, the bias toward the plus-end can be generated, on average, when KIF1A binds to the MT. We note that the same Brownian ratchet mechanism was observed in our previous MD study where the Brownian motion of myosin along the actin filament was found to be biased toward the plus-end of the actin filament in the course of the weak-to-strong binding transition [18]. Figure 3a further shows that this ratchet-like energy landscape is generated by the electrostatic interaction between KIF1A and the MT, because the ratchet-like profile almost disappeared at greater salt strengths (blue and red lines). See also Supplementary Figure S2, which directly demonstrates that the ratchet-like profile is caused by the electrostatic energy landscape. For the analysis below, we also show the snapshot structures found at the energy minima in the landscape (Figure 3b,c), where we can see that the CTT of tubulin interacts with the bound KIF1A.

### 2.3. Effect of the Electrostatic Screening

**Figure 4:**
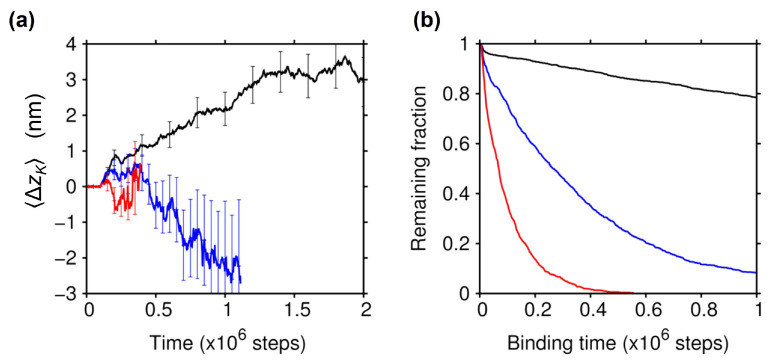
Effect of the Debye screening on biased Brownian motion. (**a**) The results of $\langle \Delta z_{K}\left(t\right)\rangle$ obtained by binding simulations with stronger electrostatic screenings of λ=0.78 nm (blue) and λ=0.67 nm (red). The average time course was terminated at the point where the number of KIF1A bound to the MT became less than 100. Error bars indicate the statistical uncertainty at the 68% confidence interval. For comparison, $\langle \Delta z_{K}\left(t\right)\rangle$ for λ=0.95 nm (the same result as in Figure 2c) is displayed (black). (**b**) The fraction of KIF1A that remained bound to the MT is shown as a function of the binding time for λ=0.95 nm (black), λ=0.78 nm (blue), and λ=0.67 nm (red).

As expected from the energy landscapes shown above, the plus-end bias observed in the Brownian motion of KIF1A when binding to the MT actually disappeared at greater salt strengths (Figure 4a), confirming the critical role of the electrostatic interaction between KIF1A and the MT in generating the bias in the Brownian motion. Under these strong screening conditions, the statistical uncertainty of $\langle \Delta z_{K}\left(t\right)\rangle$ became very large due to the reduced binding affinity of KIF1A to the MT; while the number of trajectories where the binding occurred was decreased at stronger screenings (1121 at λ=0.78 nm, and 1023 at λ=0.67 nm), the binding time was more greatly decreased as the screening was strengthened (Figure 4b). This significant reduction of binding affinity at greater salt strengths is in accord with the single molecule experiments of KIF1A [1,8,11].

### 2.4. Essential Role of the C-Terminal Tail of Tubulin

**Figure 5:**
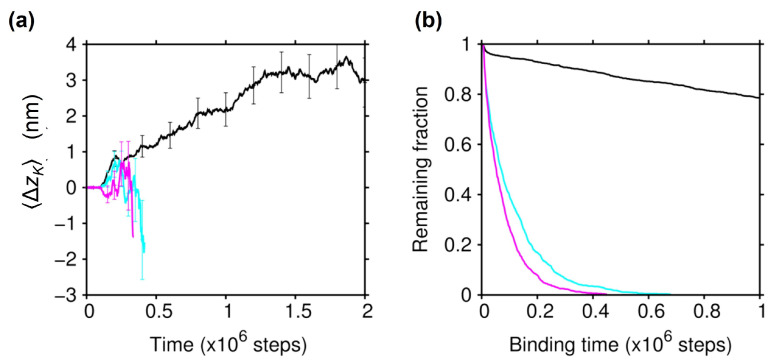
Effect of the CTT modifications on biased Brownian motion. (**a**) The results of $\langle \Delta z_{K}\left(t\right)\rangle$ obtained by binding simulations with modified tubulins where all CTTs (E-hooks) were truncated (cyan) and where all the negatively-charged residues in the CTT were neutralized (magenta). The average time course was terminated at the point where the number of KIF1A bound to the MT became less than 100. Error bars indicate the statistical uncertainty at the 68% confidence interval. For comparison, $\langle \Delta z_{K}\left(t\right)\rangle$ for wild-type tubulin (the same result as in Figure 2b) is displayed (black). (**b**) The fraction of KIF1A that remained bound to the MT is shown as a function of the binding time for the modified tubulins with the CTT truncated (cyan) and with the CTT charge neutralized (magenta).

As seen in Figure 3b,c, the CTT of tubulin interacts with KIF1A, mainly with the K-loop, in the lowest-energy KIF1A-MT bound states; the CTT of α tubulin was found to interact with the K-loop in 52% of the bound snapshots (∼130,000), and the CTT of β tubulin exhibited a greater contribution of 76%. To clarify the role of the CTT in biased Brownian motion, we carried out the binding simulations using the protofilament with modified tubulins. We first modified α and β tubulins by truncating their CTT (E-hooks). We found that the affinity of KIF1A to the MT was significantly decreased, and the plus-end bias in the Brownian motion had disappeared (cyan lines in Figure 5). We then modified the α and β tubulins by neutralizing all of the acidic residues in the CTT (seven and 10 acidic residues in the CTT for the α and β tubulins, respectively). The charge-neutralization of the CTT resulted in a significant reduction of the binding affinity and the disappearance of biased Brownian motion as well (magenta lines in Figure 5). See also Supplementary Figure S2, showing that the ratchet-like energy landscape almost disappeared upon the charge-neutralization of the CTT. These results indicate that the CTT plays an essential role in the biased Brownian motion of KIF1A along the MT through the electrostatic interaction with KIF1A.

## 3. Discussion

We showed that KIF1A exhibits biased Brownian motion as it binds to the MT. The bias was found to be generated by the ratchet-like energy landscape along the MT. It is worthwhile to emphasize that the ratchet-like energy landscape alone cannot generate the bias; the system must be out of equilibrium. In this study, the non-equilibrium condition was provided by manually moving KIF1A away from the MT at the beginning of the MD simulation. In actuality, the non-equilibrium condition was provided by the hydrolysis of ATP that KIF1A catalyzes. KIF1A strongly binds to the MT in the ATP-bound state, and on the occasion of phosphate release after ATP hydrolysis, KIF1A changes its state from the strong binding to the weak binding (or detached) state [8,9,20]. In this study, the manual moving of KIF1A away from the MT simulates the detachment of KIF1A from the MT caused by phosphate release. Note that the biased Brownian motion observed in the actomyosin motor was also based on the ratchet-like energy landscape combined with the non-equilibrium condition [18].

Our MD simulation using a residue-level coarse-grained model can reproduce the binding of KIF1A to the MT and the Brownian motion of KIF1A along MT, as was observed in the single molecule experiments [1,8,9], indicating the potential applicability of the coarse-grained model. On the other hand, we did not observe the full transition to the strong binding state. One reason is that we used the KIF1A structure in the ADP-bound state and employed the coarse-grained model that treats a protein molecule as an elastic body [21,22] and is not suitable for reproducing the structural change [20] and the dielectric response [23,24] upon ADP release. Another reason comes from the limited accuracy of the intermolecular interaction in our model; introducing the dielectric constant lowering near the hydrophobic surface [25] and the effective hydrophobic interaction [26] into the present model could facilitate the strong binding. Note however that some of the key electrostatic interactions between the charged residues outside the E-hook and K-loop regions that are considered to be important for the strong binding [27,28] were transiently observed in this study, again indicating the potential applicability of the coarse-grained model. Meanwhile, MD simulations using a more accurate all-atom model should be highly valuable to understand the full details of the KIF1A-MT binding, and some attempts have already been made and have highlighted the importance of the electrostatic interaction between KIF1A and the MT [12,29].

The C-terminal tail (CTT) was shown to play an essential role in biased Brownian motion through the electrostatic interaction with KIF1A. Then, the polyglutamylation of the CTT, one of the major post-translational modifications of the CTT, is expected to affect the motility of KIF1A on the MT, because the polyglutamylation strengthens the electrostatic interaction with KIF1A by adding negatively-charged glutamates to the CTT. The increased binding affinity and the alteration of KIF1A’s motility by polyglutamylation have actually been observed in a recent study [11]. The electrostatic effects of the polyglutamylation in the CTT can be seen for other MT-binding proteins [16,17] such as microtubule-associated proteins (MAPs) and Tau, which suggests that the polyglutamylation could regulate the axonal transport through altering the binding affinity of those MT-binding proteins that impede the motility of KIF1A [11]. Considering that KIF1A moves along the MT in the dimeric form in vivo [10], it remains unclear whether the Brownian ratchet mechanism contributes to the active transport along the MT in a cell. One possibility is that the Brownian ratchet mechanism contributes little to the active transport, and instead, the hand-over-hand mechanism in the dimeric form [7] dominates. Another possibility is that the dimeric form is advantageous for the Brownian ratchet mechanism as well because KIF1A can move over a longer distance along the MT in the dimeric form (by lowering the probability of complete detachment and diffusing away from the MT) than in the monomeric form. Further study, both experimental and computational, is needed to resolve this issue.

## 4. Methods

### 4.1. System Setup

As the base structure for the KIF1A-MT complex, we used the structure determined by electron microscopy (EM) [20] where KIF1A in the ADP-bound state interacts with the MT at the junction between α and β tubulins (PDB ID: 2HXH). To study the binding of KIF1A molecule to the MT, we constructed an MT protofilament containing 20 tubulins, which was placed in parallel to the *z*-axis (see Figure 1). The *y*-axis was set perpendicular to the surface of the MT. The origin of the coordinate system was at the mass center of KIF1A in the EM structure. The disordered regions in the EM structure (amino-acid residues 256–260 and 290–303 of KIF1A, 440–451 of α-tubulin, and 438–455 of β-tubulin) were complemented by MODELLER [30]. MD simulations, described below, were conducted by using our own code.

### 4.2. Intra-/Inter-Molecular Interactions

We used a coarse-grained model as in our previous studies where the binding of myosin to an actin filament was well reproduced [18,26]. Each amino-acid residue was treated in a coarse-grained manner as a single particle, and the intramolecular interactions between adjacent residues were represented by Hookean springs, i.e.,

(1) $$V_{\mathrm{intra}}=\sum_{j=i+1}\frac{k_{1}}{2}{\left(r_{ij}-r_{ij}^{0}\right)}^{2}+\sum_{j=i+2}\frac{k_{2}}{2}{\left(r_{ij}-r_{ij}^{0}\right)}^{2}+\sum_{\begin{array}{c} j\geq i+3\\ r_{ij}\leq 1\;\mathrm{nm} \end{array}}\frac{k_{3}}{2}{\left(r_{ij}-r_{ij}^{0}\right)}^{2},$$

where *i* and *j* denote the amino-acid residue numbers and $r_{ij}$ denotes the inter-residue distance, with $r_{ij}^{0}$ being the Cα-Cα distance between the residues *i* and *j* in the base structure. The spring constants $k_{1}$, $k_{2}$, and $k_{3}$ were set at $3.6\times 10^{3}$, $1.8\times 10^{3}$, and $3.6\times 10^{2}$ kJ/mol/nm${}^{2}$, respectively, which can reproduce the intramolecular thermal fluctuation obtained from our previous all-atom MD simulation of KIF1A [29]. To consider the large flexibility of the disordered regions in the MT-interacting loops (L11 and K-loop) [20], we replaced Equation (1) by,

(2) $$\begin{array}{ccc} V_{\mathrm{loop}} & = & \sum_{j=i+1}\frac{k_{1}}{2}{\left(r_{ij}-r_{ij}^{0}\right)}^{2}\\ & + & \sum_{i}\left[\frac{f_{\alpha }\left(\theta_{i}\right)+f_{\beta }\left(\theta_{i}\right)}{2}-\sqrt{\frac{{\left(f_{\alpha }\left(\theta_{i}\right)-f_{\beta }\left(\theta_{i}\right)\right)}^{2}}{4}+\Delta^{2}}\right]+\sum_{|j-i|>3}u\left(r_{ij}\right), \end{array}$$

where the second term represents a double-well angular potential [26] for the residues in the loop regions, with $\theta_{i}$ denoting the angle formed by the adjacent residues; $f_{\alpha }\left(\theta \right)=a_{\alpha }{\left(\theta -\theta_{\alpha }\right)}^{2}+\delta$ and $f_{\beta }\left(\theta \right)=a_{\beta }{\left(\theta -\theta_{\beta }\right)}^{2}$, with $\theta_{\alpha }=1.51$, $\theta_{\beta }=2.35$, $a_{\alpha }=857$, δ=90, $a_{\beta }=41$, and Δ=50, (the angular unit is radians, and the energy unit is kJ/mol). These parameters were determined so as to reproduce the potential of the mean force obtained by the all-atom MD simulation [29]. $k_{1}$ was set to $2.1\times 10^{4}$ kJ/mol/nm${}^{2}$, and the third term in Equation (2) represents the intramolecular Coulomb and van der Waals interactions (see below) involving the residues in the loop regions. $V_{\mathrm{loop}}$ was also applied to the disordered region of the C-terminal tail (CTT) of tubulins, with the remaining residues in the tubulin protofilament kept fixed in space.

For the intermolecular interaction between KIF1A and tubulins, we considered the Coulomb and van der Waals potentials [18],

(3) $$V_{\mathrm{inter}}=\sum_{i,j}Aq_{i}q_{j}e^{-r_{ij}/\lambda }/4\pi \varepsilon r_{ij}+\sum_{i,j}B\left[{\left(r_{0}/r_{ij}\right)}^{12}-2{\left(r_{0}/r_{ij}\right)}^{6}\right].$$

The first term in Equation (3) is the Coulomb interaction with Debye–Hückle screening, where water and the counterion are considered implicitly through the dielectric constant and the Debye screening length. $q_{i}$ is the charge of the residue *i* (−1 for Asp and Glu, +1 for Lys and Arg, and +0.5 for His). *A* is the Coulomb constant (140 kJ/mol·nm). ε is the dielectric constant of water. λ is the Debye screening length. In this study, ε was set at 74 (at 310 K) and λ at 0.95 nm (corresponding to the salt strength of 100 mM), unless otherwise noted. The second term in Equation (3) represents the van der Waals interaction, for which we used the 6–12-type Lennard–Jones (LJ) potential, with $r_{0}=0.8$ nm and B=0.1 kJ/mol. The Coulomb and van der Waals interactions were truncated at 7 nm and 1.5 nm, respectively. These potentials were also used for the third term in Equation (2).

### 4.3. Molecular Dynamics

To simulate the binding of KIF1A to MT, we initially moved KIF1A away from the MT by 6 nm. We prepared uniformly-distributed initial *z*-positions of KIF1A along the MT that cover the entire period of the tubulin protofilament (8 nm). To keep KIF1A from diffusing laterally (i.e., in the *x*-direction), we restricted the lateral movement of KIF1A by restraining potential $\sum_{i}\frac{k_{r}}{2}{\left(x_{i}-x_{i}^{0}\right)}^{2}$, where $k_{r}=0.36$ kJ/mol/nm${}^{2}$ and $x_{i}^{0}$ denotes the initial *x*-position of the residue *i* in KIF1A. The Langevin equation was used for the molecular dynamics simulation, with the mass for the coarse-grained particle set at $1.8\times 10^{-25}$ kg and the temperature at 310 K. As in our previous study [18], we used a low viscosity so that the frictional coefficient for the center of mass of KIF1A becomes $2.0\times 10^{-13}$ kg/s (corresponding to the diffusion coefficient *D* of 22 nm${}^{2}$/ns), which enhances the translational diffusion of KIF1A by ∼100-fold compared to that in water. The equation of motion was numerically integrated with the time increment of 3.3 × 10${}^{-5}$ ns, and 2 × 10${}^{6}$ step integration was carried out. For each initial *z*-position of KIF1A, we conducted 300 independent MD runs (total 2400 runs) under each specified condition; we used different random number seeds for the fluctuation force term in the Langevin equation and thermally equilibrated the system for the initial 0.1 × 10^6^ steps by restraining KIF1A at each initial position.

## Data Availability

Data is contained within the article and Supplementary Materials.

## Supplementary Materials

The following are available online at https://www.mdpi.com/1422-0067/22/4/1547/s1: Figure S1: Influence of the restraint applied to KIF1A, Figure S2: Electrostatic energy landscape for the KIF1A-MT interaction.
