# Supplementary material for: Biased Brownian Motion of KIF1A and the Role of Tubulin’s C-Terminal Tail Studied by Molecular Dynamics Simulation
Source: Int J Mol Sci. 2021 Feb 4;22(4):1547. doi: 10.3390/ijms22041547 (PMC7913626; doi:10.3390/ijms22041547)
Supplement: Supplementary file 1 [file ijms-22-01547-s001.pdf]

Biased Brownian Motion of KIF1A and the Role of Tubulin's C-Terminal Tail Studied by Molecular Dynamics Simulation (Supplementary Figures)

Yukinobu Mizuhara[1] and Mitsunori Takano*[1]

[1] *Department of Pure and Applied Physics, Waseda University, Ohkubo 3-4-1, Shinjuku-Ku, Tokyo 169-8555, Japan*

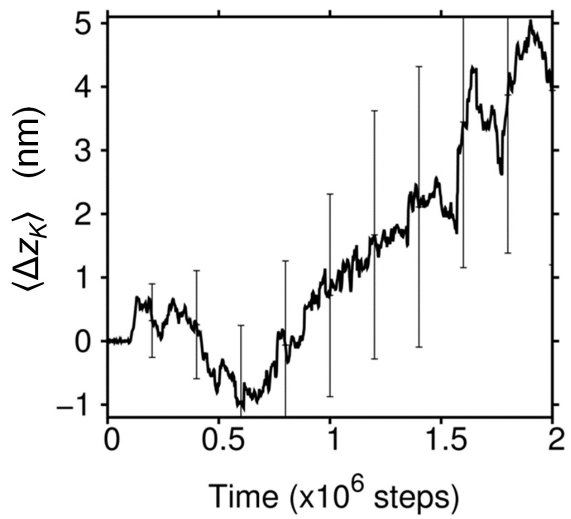

FIG. S1. To see the influence of the restraint applied to KIF1A (see Methods), which was introduced to keep KIF1A from diffusing laterally (in the x direction), we examined another restraint potential in the x direction that acts on the mass center of KIF1A in the form of $\frac{1}{2}Nk_r(x_K - x_K^0)^2$, with x_K denoting the x-position of the mass center of KIF1A. Ensemble average of the z-displacement of KIF1A is shown as a function of time, which was calculated from the trajectories where KIF1A binding was observed (total 1348 runs out of 2400). Error bars indicate the statistical uncertainty (the standard error of the mean) at 68% confidence interval.

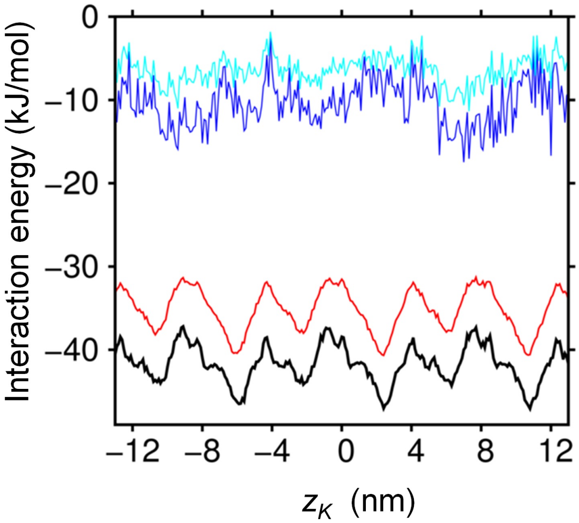

FIG. S2. Electrostatic energy landscape for the KIF1A-MT interaction. Average electrostatic interaction energy between KIF1A and MT is shown as a function of z_K (red). The result for the total KIF1A-MT interaction energy (black; the same data as in Fig. 3 in the main text) is also included for comparison. The energy landscapes for the electrostatic interaction between KIF1A and MT where CTT of tubulins were charge-neutralized are shown (cyan) together with the landscape for the total interaction energy (blue).
